# On table POCUS assessment for the IVC following abdominal packing: how I do it

**DOI:** 10.1186/s13017-016-0092-3

**Published:** 2016-08-05

**Authors:** Fikri M. Abu-Zidan

**Affiliations:** Department of Surgery, College of Medicine and Health Sciences, UAE University, Al-Ain, United Arab Emirates

**Keywords:** Abdomen, Point-of-care ultrasound, IVC, Packing

## Abstract

**Background:**

Some surgeons may lack proper experience in abdominal packing. Overpacking may directly compress the inferior vena cava (IVC). This reduces the venous return and possibly causes hypotension. Here, a new on table Point-of-Care Ultrasound application that has been recently used to assess the effect of abdominal packing on the IVC diameter is described. Following abdominal packing, a small print convex array probe with low frequency (2–5 MHz) is used to visualize the IVC. Using the B mode, the IVC can be directly evaluated through a hepatic window between the ribs. The ultrasound beam should be vertical to the IVC longitudinal section at its midpoint. The abdominal towels will be in front of the IVC. This will enable us to judge whether there was overpacking on the IVC.

**Results:**

Our method demonstrates that overpacking does not compress the IVC in a patient whose blood pressure has improved. The IVC diameter progressively increases on table and in the ICU with active resuscitation implying that bleeding stopped and the resuscitation was successful. Furthermore, presence of intra-peritoneal fluid can be excluded.

**Conclusions:**

This new application of ultrasound evaluation of IVC patency after abdominal packing is simple, practical, easily reproducible, and can guide a less experienced surgeon in determining if overpacking of the abdomen is the cause of hypotension. Ultrasound findings should be correlated with the clinical picture to be useful.

## Background

Abdominal packing is a useful simple technique which is used in damage control surgery (DCS) to stop severe bleeding in multiply injured patients who are acidotic, hypothermic and coagulopathic [1]. This stabilises the patient and gives time to restore the physiological derangement through damage control resuscitation [2]. Finally, the patient can be timely reoperated upon to restore the functional anatomy [3]. In addition, surgeon-performed Point-of-Care Ultrasound (POCUS) has become an important critical decision making tool in managing critically-ill patients [4, 5]. Both DCS and POCUS have been used successfully in the military and prehopsital settings [3, 6].

Some surgeons may lack proper experience in abdominal packing and possibly overpack the abdomen. Overpacking has side effects. The packs and its associated increased intra-abdominal pressure may directly compress the inferior vena cava (IVC). This reduces the venous return and may cause hypotension. Furthermore, transportation time can be long before arriving to a proper hospital. It is therefore reasonable to aim at proper intra-abdominal packing that stops bleeding but without obliterating the IVC. Sonographic measurement of the IVC diameter has been recently advocated in the evaluation of patients in hemorrhagic or septic shock [7, 8]. POCUS machines are now usually available in surgical theatres because they are used routinely in inserting sonographic guided central lines. It would be then attractive to evaluate the IVC patency by POCUS following abdominal packing for DCS. The author of the present paper has recently reviewed the literature on the use of ultrasound measurement of IVC diameter in resuscitation [7]. The relative change of IVC diameter in trauma patients could differentiate between proper resuscitation responders from transient responders who develop recurrent shock [9]. IVC measurement was feasible in 92 % of patients having septic shock [10]. Using ultrasound in evaluating the IVC following abdominal packing was not described before. The present paper describes a recent on table POCUS application that can be used to assess the effect of abdominal packing on the IVC diameter following DCS.

## Methods

**Fig. 1:**
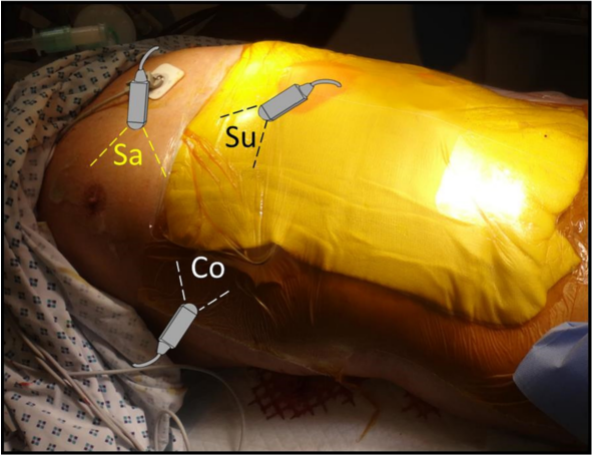
There are three windows in which we can measure the IVC: The subxiphoid (Su), the direct sagittal (Sa), and the coronal view (Co). The only useful approach after DCS is the direct sagittal approach

**Fig. 2:**
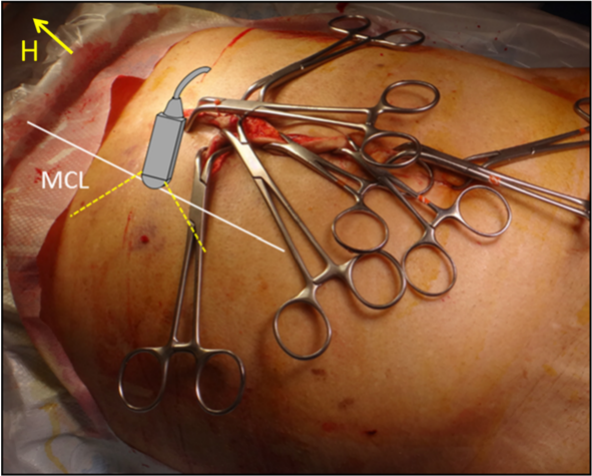
A diagram demostrating the technique to visualize the IVC by ultrasound in a longitudinal section following damage control surgery with abdominal packing. The skin was closed with towel clips following abdominal packing without closure of the fascia. A small print convex array probe with low frequency (2–5 MHz) should be used. The probe is located at the right mid-clavicular line (MCL) at the lower chest wall vertical to the skin. H = location of the head

**Fig. 3:**
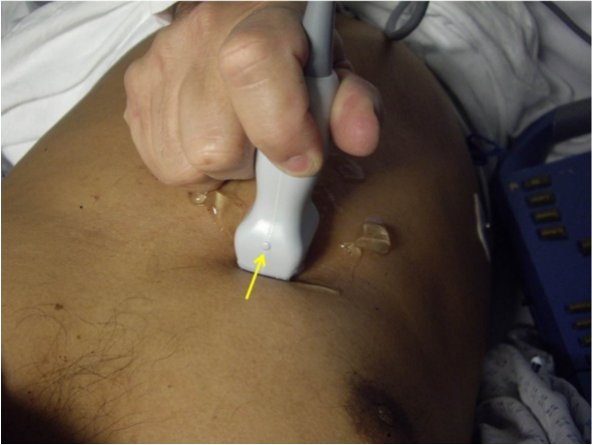
The marker of the probe should point proximally towards the head of the patient (arrow). It may be required to tilt the probe a little towards the right shoulder to be parallel to the IVC

A small print convex array probe with low frequency (2–5 MHz) should be used. Ultrasound waves are generated perpendicular to the surface of this probe. This probe has deep sonographic penetration and wide view. It will enable sonographic visualization of the IVC between the ribs and through a hepatic window to directly measure the antero-posterior section of the IVC [11]. There is usually no intra-peritoneal sonographic air barrier hindering the view if this approach was used. The probe should be covered by sterile gel and plastic sleeve. Sufficient sterile gel should be used to have proper contact with the skin so that the air does not disturb the view. There are three windows in which we can visualize the IVC by ultrasound: the subxiphoid, the direct sagittal and the coronal view (Fig. 1). The marker of the probe should point proximally towards the head. This will be on the right side of the ultrasound screen. The probe is located at the right mid-clavicular line, at the lower chest wall, and vertical to the skin (Fig. 2). It is then *shifted* in both lateral directions to locate the IVC. Gentle slow lateral *fanning* movements may be needed to locate the IVC. It may be required to *tilt* the probe a little towards the right shoulder to be parallel to the IVC (Fig. 3). The commonly used subxiphoid view cannot be used following damage control laparotomy for different reasons. *First*, the midline laparotomy and its dressing will be in the way. *Second*, the presence of distended stomach and free intra-peritoneal air will hinder the view, and *third*, the abdomen may be left open. The coronal section will cut the IVC transversely and will not measure the antero-posterior diameter of the IVC.

**Fig. 4:**
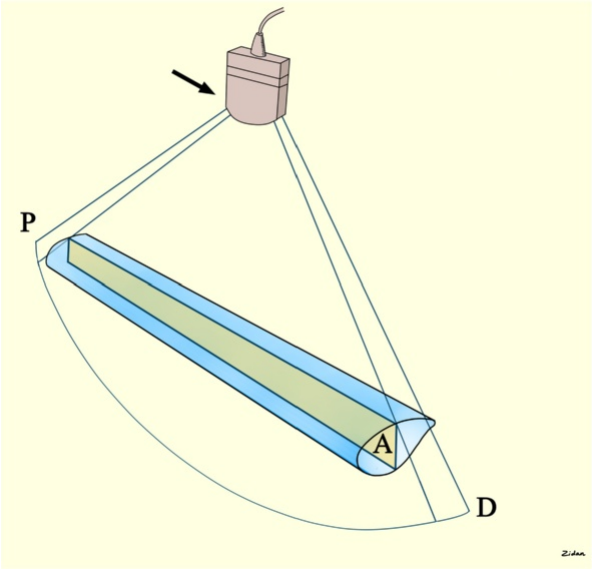
The ultrasound beam should be vertical to the IVC longitudinal section (A) at its midpoint. P = proximal, D = distal, Black arrow = marker of the probe

**Fig. 5:**
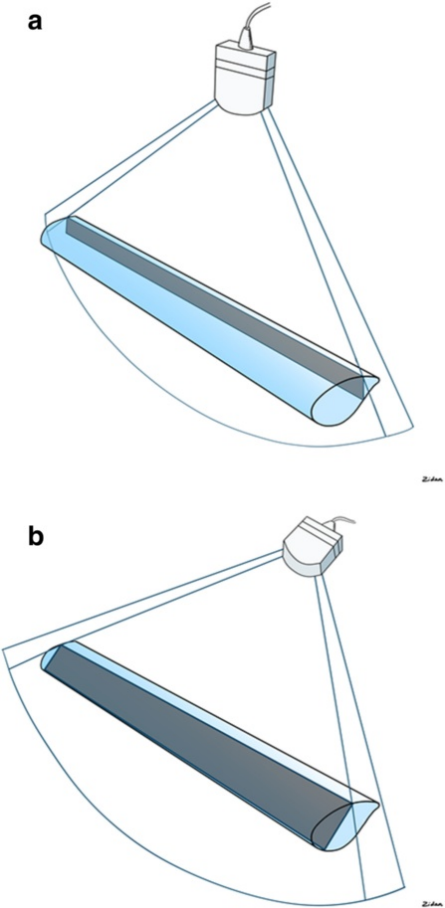
The two common pitfalls in visualizing the IVC by ultrasound are cutting it at the periphery **a** giving a false impression that it is small **a** or cutting it obliquely giving a false impression that it is large **b**

**Fig. 6:**
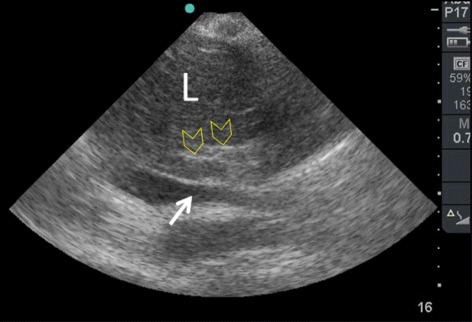
Using the hepatic window, a two dimensional B mode image shows that the IVC was patent (white arrow). The abdominal towels appear as whitish linear layers without shadowing (yellow arrow heads) anterior to the IVC. L = liver

Using the B mode, the IVC can be evaluated in its longitudinal diameter. The ultrasound beam should be vertical to the IVC longitudinal section at its midpoint (Figs. 4 and 5). The IVC will be black in color on the B mode ultrasound image. The abdominal towels will appear as whitish linear layers without shadowing (behaving like a fibrous tissue) anterior to the IVC. This will show whether the IVC is compressed or not (Fig. 6).

## Results

**Fig. 7:**
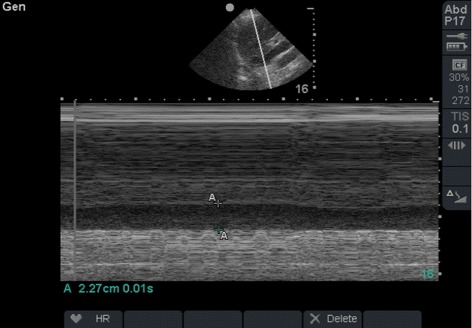
A two dimensional B mode image performed in the ICU 24 hours later during damage control resuscitation shows that the IVC diameter (A-A) increased to 2.27 cm. The patient had a blood pressure of 130/80 mmHg and had adequate urine output. Hypothermia, acidosis, and coagulopathy were corrected at this stage

Our method serves to assess excessive pressure on the IVC in abdominal packing (Fig. 6). The IVC diameter progressively increases on table and in the ICU with active resuscitation implying that bleeding stopped and the resuscitation was successful (Fig. 7). Furthermore, presence of intra-peritoneal fluid can be detected.

## Discussion

The value of POCUS in the management of patients in hemorrhagic or septic shock using specific protocols is well-established [5, 8]. Our group routinely follows the Rapid Ultrasound in Shock (RUSH) protocol which examines the pump (heart), tubes (great vessels) and reservoir (free intra-peritoneal or intra-thoracic fluid) [5]. Performing an on table RUSH protocol directly following DCS is very attractive. Using the same approach, it would be logical to evaluate the IVC patency by POCUS following abdominal packing. POCUS is quick, done bedside and does not interfere with reuscitation. It may yield very useful information during the continuation of damage control resuscitation on the operating table after the damage control laparotomy and before transferring the patient to the ICU.

The skin was closed with towel clips following abdominal packing without closure of the fascia in the patient shown in Fig. 2 because we were anticipating to close the fascia within 48 hours without the development of postoperative abdominal hypertension. There are different methods for temporary abdominal closure that has been recently detailed in a position paper published by the World Society of Emergency Surgery [12]. Advantages and disadvantages of each of these techniques were described in detail in that paper which is beyond the scope of the present communication. These techniques included the plastic silo, negative pressure therapy (NPT), and the combined NPT with fascial approximation. The most extensively used method is NPT while the most promising one is the combined NPT with fascial approximation. Using our described ultrasound approach, POCUS can be applied with all these different techniques of closing the abdomen because a transthoracic hepatic ultrasound window is away from the wound.

Sonographic appearance of intra-peritoneal free air has been described in detail [13]. They result from reflections of the ultrasound waves at the interface between the soft tissue and air. This is accompanied by a reverberation artefact. This artefact typically appears as increased echogenicity of a peritoneal stripe accompanied by posterior reverberation parallel echogenic lines having equal distances that will hide the organs. This image can be changed by changing the patient's position. The echogenic appearance of the intra-abdominal towels (as shown in Fig. 6) is different. The echogenic lines are not parallel, not of the same length, not at equal distances, and finally they do not hide the liver. Although these ultrasound fine details can be observed by an experienced sonographer, they are not needed for evaluating the IVC diameter which can be done by a less experienced sonographer.

Weekes et al. [14] prospectively studied trauma patients and found that gross appearance of the IVC size agreed with the actual measured IVC diameter during resuscitation. A meta-analysis, which included a total of 86 hypovolemic patients and 189 controls, found that IVC diameter was significantly less in hypovolemic patients compared with controls [15]. A recent prospective randomized controlled trial in injured patients having hypotension or tachycardia treated in a Level I trauma center [16] found that focused ultrasound studies of the heart and IVC significantly reduced the IV fluid requirements and the time to surgery. We have recently reported that increased intra-abdominal pressure in abdominal compartment syndrome causes direct pressure on the IVC which reduces the antero-posterior IVC diameter [17].

Our method is logically more useful when the blood pressure of the patient improves. Its value may be questionnable when the patient significantly continues to bleed because the sonographic IVC diameter will be small. Nevertheless, we have to clarify that active intra-abdominal arterial bleeding does not respond to packing and should be actually dealt with surgically. However, on table POCUS is useful in detecting an on going intraperitoneal bleeding. In a patient with improving blood pressure, the IVC diameter will increase indicating that IVC was not overpacked, bleeding stopped, and the resuscitation was successful.

## Conclusions

This new application of ultrasound evaluation of IVC patency after abdominal packing is simple, practical, easily reproducible, and can guide a less experienced surgeon in determining if overpacking of the abdomen is the cause of hypotension. Ultrasound findings should be correlated with the clinical picture to be useful.

## Abbreviations

IVC: Inferior vena cava
POCUS: Point-of-care ultrasound
ICU: Intensive care unit
DCS: Damage control surgery

## References

[1] Bashir MM, Abu-Zidan FM (2003). Damage control surgery for abdominal trauma. Eur J Surg.

[2] Kaafarani HM, Velmahos GC (2014). Damage control resuscitation in trauma. Scand J Surg..

[3] Blackbourne LH (2008). Combat damage control surgery. Crit Care Med.

[4] Abu-Zidan FM (2012). Point-of-care ultrasound in critically ill patients: Where do we stand?. J Emerg Trauma Shock..

[5] Perera P, Mailhot T, Riley D, Mandavia D (2010). The RUSH exam: rapid ultrasound in SHock in the evaluation of the critically ill. Emerg Med Clin North Am..

[6] Dittrich K, Abu-Zidan FM (2004). Role of ultrasound in mass-casualty situations. Int. J. Disaster Med..

[7] Abu-Zidan FM (2016). Optimizing the value of measuring inferior vena cava diameter in shocked patients. World J Crit Care Med.

[8] Seif D, Mailhot T, Perera P, Mandavia D (2012). Caval sonography in shock: a noninvasive method for evaluating intravascular volume in critically ill patients. J Ultrasound Med.

[9] Yanagawa Y, Sakamoto T, Okada Y (2007). Hypovolemic shock evaluated by sonographic measurement of the inferior vena cava during resuscitation in trauma patients. J Trauma..

[10] Coen D, Cortellaro F, Pasini S, Tombini V, Vaccaro A, Montalbetti L, Cazzaniga M, Boghi D (2014). Towards a less invasive approach to the early goal-directed treatment of septic shock in the ED. Am J Emerg Med..

[11] Abu-Zidan FM, Hefny AF, Corr P (2011). Clinical ultrasound physics. J Emerg Trauma Shock.

[12] Sartelli M, Abu-Zidan FM, Ansaloni L (2015). The role of the open abdomen procedure in managing severe abdominal sepsis: WSES position paper. World J Emerg Surg.

[13] Hefny AF, Abu-Zidan FM (2011). Sonographic diagnosis of intraperitoneal free air. J Emerg Trauma Shock..

[14] Weekes AJ, Tassone HM, Babcock A, Quirke DP, Norton HJ, Jayarama K, Tayal VS (2011). Comparison of serial qualitative and quantitative assessments of caval index and left ventricular systolic function during early fluid resuscitation of hypotensive emergency department patients. Acad Emerg Med.

[15] Dipti A, Soucy Z, Surana A, Chandra S (2012). Role of inferior vena cava diameter in assessment of volume status: a meta-analysis. Am J Emerg Med.

[16] Ferrada P, Evans D, Wolfe L, Anand RJ, Vanguri P, Mayglothling J, Whelan J, Malhotra A, Goldberg S, Duane T, Aboutanos M, Ivatury RR (2014). Findings of a randomized controlled trial using limited transthoracic echocardiogram (LTTE) as a hemodynamic monitoring tool in the trauma bay. J Trauma Acute Care Surg..

[17] Abu-Zidan FM, Idris K (2015). Sonographic measurement of the IVC diameter as an indicator for fluid resuscitation: beware of the intra-abdominal pressure. World J Surg.

[18] Abu-Zidan FM, Idris K, Khalifa M (2016). Strangulated epigastric hernia in a 90-year-old man: Point-of-Care Ultrasound (POCUS) as a saving kit: case report. Int J Surg Case Rep..

